# OxyR contributes to the oxidative stress capacity and virulence of hypervirulent *Klebsiella pneumoniae* ATCC 43816

**DOI:** 10.3389/fcimb.2025.1661384

**Published:** 2026-01-07

**Authors:** Ruxia Zhang, Yu Zheng, Chao Ding, Jiayu Wu, Wanqiu Zhu, Xiaojue Zhu, Guoxin Xu, Long Chen

**Affiliations:** Department of Clinical Laboratory, The First People’s Hospital of Zhangjiagang City, Zhangjiagang Hospital Affiliated to Soochow University, Zhangjiagang, Jiangsu, China

**Keywords:** gene regulation, hypervirulent *Klebsiella pneumoniae*, oxidative stress, OxyR, virulence

## Abstract

**Introduction:**

Hypervirulent *Klebsiella pneumoniae* (hvKP) is an emerging pathogen associated with severe invasive infections and high mortality, in which resistance to host-derived reactive oxygen species (ROS) is critical for immune evasion and persistence. However, the mechanisms underlying oxidative stress resistance in hvKP remain poorly understood, and the role of the global regulator OxyR in this species has not been fully elucidated.

**Methods:**

In this study, VK055_RS16305 was first identified as an OxyR homologue in *K. pneumoniae* ATCC 43816 by sequence alignment. The *oxyR* deletion mutant was generated using a CRISPR/Cas9-based genome editing system, whereas the complemented strain was obtained using the pSTV28 plasmid carrying *oxyR*. We then compared their growth characteristics, susceptibility to H₂O₂, biofilm formation, and virulence in *Galleria mellonella* and mouse infection models, and performed RNA sequencing followed by qRT-PCR to characterize the OxyR regulon under oxidative stress.

**Results:**

Deletion of *oxyR* did not alter bacterial growth or colony morphology under non-stress conditions, but markedly increased susceptibility to H₂O₂ and impaired biofilm formation. *In vivo*, the *oxyR* mutant exhibited attenuated virulence, with improved survival of *Galleria mellonella* and mice and significantly reduced bacterial burdens in blood, liver, lung, and spleen, all of which were restored by genetic complementation. Transcriptomic analysis revealed that OxyR positively regulates multiple oxidative stress–associated genes, including *hemH, grxA, gsk, katG*, and *ahpC*, in response to H₂O₂.

**Discussion:**

Together, these findings demonstrate that OxyR is a key regulator of oxidative stress defense, biofilm formation, and systemic virulence in hvKP, providing new insight into OxyR-mediated pathogenic mechanisms in *K. pneumoniae*.

## 1 Introduction

*Klebsiella pneumoniae* is an Enterobacteriaceae bacterium commonly found in humans (Huang et al., 2022); it is a conditionally pathogenic bacterium that can invade multiple tissues to cause pneumonia, meningitis, bloodstream infections, and urinary tract infections, particularly in hospitalized or immunocompromised individuals, although infections also occur in otherwise healthy hosts (Li et al., 2014; Vuotto et al., 2017; Guerra et al., 2022). Following the 1980s report of *K. pneumoniae*-associated liver abscess complicated by metastatic endophthalmitis (Liu et al., 1986), this pathogen was categorized into classic *K. pneumoniae* (cKP) and hypervirulent *K. pneumoniae* (hvKP) (Paczosa and Mecsas, 2016). Compared with cKP, hvKP has greater pathogenicity and a stronger ability to spread (Russo and Marr, 2019), posing a serious threat to public health (Zhu et al., 2021). The mechanisms underlying the regulation of the virulence and pathogenesis of hvKP remain unclear.

Innate immunity is the first barrier encountered by pathogenic microorganisms invading the body. Innate immunity defense includes physical barriers (such as the skin and mucosa), cells (such as macrophages and neutrophils), and chemical mediators (including ROS and antimicrobial peptides) (Kaur and Secord, 2021). Macrophages and neutrophils recognize and engulf invading pathogenic bacteria, producing ROS and other cytokines to attack the bacteria (Thi et al., 2012). The ROS production can directly damage the cell wall, membrane, and DNA of bacteria, thereby destroying invading bacteria (Underhill and Ozinsky, 2002). To survive, pathogenic bacteria have developed a variety of mechanisms to resist the host immune system. For example, the capsule of *K. pneumoniae* can effectively block direct attack by ROS and protect the bacterium from phagocytic cells (such as macrophages) (Wanford et al., 2021; Liang et al., 2023). Many pathogens produce enzymes (such as superoxide dismutase and catalase) and small-molecule antioxidants (such as glutathione) that neutralize ROS and reduce the associated damage (Wang et al., 2024). Some bacteria (e.g., *Campylobacter jejuni*) can survive inside the host cell and thus avoid attack by the immune system (Ó Cróinín and Backert, 2012; Kemper and Hensel, 2023). Moreover, global regulators such as OxyR mediate cellular detoxification of harmful oxidizing agents (Hou et al., 2019).

As a classic global response oxidative stress response regulator, OxyR has been widely studied in gram-negative bacteria, such as *E. coli* and *Salmonella* (Méndez et al., 2022; Tristano et al., 2023), but little is known about its role in *K. pneumoniae*. OxyR belongs to the LysR family and is one of the major transcriptional regulators involved in the defense against oxidative stress as an activator of genes encoding peroxidative detoxification enzymes. The OxyR protein is a homotetramer that contains two conserved cysteine residues in each subunit. Under oxidative stress, the structural change in the homotetramer may be caused by the formation of a disulfide bond between the two conserved cysteine residues. This change may alter DNA binding properties and result in an efficient interaction between OxyR and RNA polymerase (Choi et al., 2001; Paget and Buttner, 2003; Cappelli et al., 2022). When *E. coli* is exposed to H_2_O_2_, the expression of GrxA, TrxC, and KatG is regulated by OxyR and plays a role in the neutralization of ROS (Ritz et al., 2000; Fernandes and Holmgren, 2004; Gutierrez-Ríos et al., 2007; Hou et al., 2019). In *Salmonella*, OxyR senses oxidative stress and participates in the regulation of peroxide metabolism and the expression of oxidative stress defense-related genes, which are critical for the survival of *Salmonella* in a strongly oxidizing environment. In addition, OxyR negatively regulates H3-T6SS and its effector protein TepB in *P. aeruginosa* PA14, enhances bacterial resistance to multiple stresses, promotes biofilm formation and motility, and enhances pathogenicity (Yang et al., 2022). OxyR promotes pathogenesis by promoting biofilm formation, fimbrial expression, and mucosal colonization in other pathogenic bacteria (Shin et al., 2020; Cappelli et al., 2022; Hancock et al., 2022). However, the regulation and function of OxyR in *K. pneumoniae*, especially in hvKP, are still poorly understood. Therefore, in the present study, we characterized an OxyR ortholog in the hypervirulent *K. pneumoniae* strain ATCC 43816 and constructed a deletion mutant and corresponding complement strain to provide materials for exploring the biological role of *oxyR* in *K. pneumoniae* ATCC 43816.

## 2 Materials and methods

### 2.1 Bacterial strains and growth conditions

The ST493/KL2 highly virulent *K. pneumoniae* ATCC 43816 strain was obtained from the American Type Culture Collection (ATCC) and used as the wild-type strain. The *E*. *coli* DH5α and *K. pneumoniae* strains were cultured aerobically in lysogeny broth (LB) medium (1% tryptone, 0.5% yeast extract, and 1% NaCl). Strains with the temperature-sensitive plasmid pCasKP were cultured at 30°C. Antibiotics were selectively added at the following concentrations: rifampicin, 50 mg/mL; apramycin, 30 mg/mL; ampicillin, 100 mg/mL; and chloramphenicol, 20 mg/mL. The primers used in this study are shown in **Supplementary Table S1** (**Supplementary Material**). The strains and plasmids used in this study are listed in **Supplementary Table S2** (**Supplementary Material**).

### 2.2 Construction of *oxyR* mutant

The *K. pneumoniae* mutant was constructed as previously described (Lv et al., 2022). Briefly, the CRISPR/Cas9-mediated genome editing system was utilized to build the *oxyR* mutant (Wang et al., 2018). The pCasKP plasmid was transferred via electroporation into wild-type receptor cells. After incubation at 30°C for 16 h, the colonies were subjected to verification by PCR and electrophoresis. The 20 nt base-pairing region (N20) of a sgRNA was designed via an online tool (http://crispr.dfci.harvard.edu/SSC). The primers N20-F and N20-R were used to produce the sgRNA fragment by annealing using Annealing Buffer for DNA Oligos (Beyotime, China). The plasmid pSGKP was digested with *Bsa*I and then ligated with the N20 fragment using T4 ligase to construct the recombinant pSGKP-*oxyR*-N20. Linear homologous DNA fragments served as repair templates and were coelectrotransformed with pSGKP-*oxyR*-N20 into ATCC 43816 harboring pCasKP induced with L-arabinose. The strains were selected on LB agar plates containing 30 mg/mL ampicillin and 100 mg/mL rifampicin and then subjected to PCR and sequencing for verification. The *oxyR* mutant strain derived from ATCC 43816 was named *ΔoxyR*.

### 2.3 Complementation of *oxyR*

To generate the OxyR complementation vector, the pSTV28 plasmid was used. The *oxyR* gene was amplified from the ATCC 43816 genome using *oxyR*-CF and *oxyR*-CR primers. The PCR products were gel-purified and then ligated into the multiple cloning site of pSTV28. The recombinant plasmid pSTV28-*oxyR* was transformed into *E. coli* DH5α and further verified by PCR and sequencing. To obtain the OxyR complement strain, pSTV28-*oxyR* was electrotransformed into the mutant strain *ΔoxyR* (named *oxyR*-C). The wild-type strain (WT) and mutant strain (*ΔoxyR*) were transformed with the empty vector pSTV28 as a control, and the resultant strains were named WT-pSTV28 and *ΔoxyR*-pSTV28, respectively.

### 2.4 Bacterial growth curves

The growth curves of the *K. pneumoniae* wild-type strain and derivatives were prepared via passaging and growth in LB broth. The overnight bacterial cultures were transferred to 20 mL of fresh LB broth at a ratio of 1:100 and then incubated at 37°C with shaking at 200 rpm. OD_600_ value was measured at one-hour intervals. The experiment was repeated three times, and all the samples were measured in triplicate. To detect growth under various H_2_O_2_ concentrations, bacterial cultures were grown to the exponential phase (OD_600_ = 0.4). In order to elicit oxidative stress without causing extensive killing, the concentration of 2 mM H_2_O_2_ was chosen. H_2_O_2_ (3%) was added to the medium to achieve a final concentration of 2 mM. The growth curve was recorded as described above. For statistical inference, we pre-specified the endpoint at 24 h and calculated the area under each biological replicate’s growth curve (AUC) over 0–24 h using the trapezoidal rule in GraphPad Prism. Group comparisons of AUC and endpoint OD_600_ were performed by the Kruskal-Wallis test followed by Dunn’s multiple comparisons test.

### 2.5 H_2_O_2_ sensitivity test

A disk diffusion assay was used to test the H_2_O_2_ sensitivity. For details, overnight bacterial cultures were transferred into 5 mL of fresh LB broth at a ratio of 1:100 and incubated until the OD_600_ value reached 1.0. The agar diffusion test plate was prepared as follows: the lower layer consisted of 20 mL LB solid medium, and the upper layer consisted of 5mL LB soft solid medium plus 200 μL of the above-mentioned bacterial suspension. A piece of a clean and sterile disk was attached to the middle of the plate, and then 5 μL of 3% H_2_O_2_ solution was added. After incubation at 37°C for 24 hours, the diameters of the inhibition zones were measured. The test was repeated three times, and all the samples were measured in triplicate.

### 2.6 Biofilm formation assay

Bacterial biofilm formation was estimated by a crystal violet (CV) assay performed as previously described (Khajanchi et al., 2012; Fu et al., 2018). *K. pneumoniae* strains were grown in LB broth overnight and transferred at a ratio of 1:100 into 20 mL of fresh LB broth for subculture. When the bacterial culture reached the late logarithmic phase (OD_600_ = 1.0), 20 μL of culture was transferred to a 96-well polystyrene microtiter plate containing 180 μL of fresh LB broth per well. After incubation at 37°C for 42 h, the medium inside the wells was removed, and the plate was washed gently three times to remove the planktonic cells. Samples were fixed with formaldehyde for 20 min and then stained by adding 250 μL of 1% crystal violet (Beyotime, China) for 30 min. The stained area was dissolved by 33% glacial acetic acid. The biofilm formation was quantified by measuring the absorbance value at 595 nm. The absorbance data were obtained from three replicate experiments.

### 2.7 RNA sequencing and differential expression analysis

Overnight bacterial cultures were transferred at a ratio of 1:100 into fresh LB broth and grown to an OD_600_ of 0.4. The bacteria were collected by centrifugation and resuspended in LB broth containing 1 mM H_2_O_2_ to elicit stress response. After incubation for 30 min, bacteria were collected through centrifugation and subjected to total RNA extraction using the RNeasy kit (Qiagen, Germany). DNase was added to eliminate DNA contamination. The RNA-sequencing was performed by Majorbio Bio-pharm Technology Co., Ltd (Shanghai, China). RNAseq libraries were prepared using the TruSeqTM RNA sample preparation Kit (Illumina, United States). Ribosomal RNA (rRNA) was removed using the RiboCop rRNA Depletion Kit for Mixed Bacterial Samples (Lexogen, United States). Paired-end RNA sequencing was performed with the Illumina Novaseq Xplus. All analyses were performed using the Majorbio Cloud Platform (www.majorbio.com). Quantitative reverse transcription PCR (qRT-PCR) was used to measure the transcription levels of target genes. Power SYBR Green Master Mix (ABI, United States) was used according to the manufacturer’s instructions. Differences in gene expression were normalized to 16S rRNA expression and calculated by the 2^-ΔΔCt^ method. All qRT-PCR assays were repeated at least three times.

### 2.8 *Galleria mellonella* killing assay

*Galleria mellonella* larval infection modeling is considered an effective method for assessing the pathogenicity of *K. pneumoniae* (Insua et al., 2013; Gu et al., 2018; Lv et al., 2022). *G. mellonella* larvae (approximately 35 days post-hatch, weighing 300 ± 50 mg) were purchased from Tianjin Huiyude Biotech Company, Tianjin, China. The virulence of WT-pSTV28, *ΔoxyR*-pSTV28, and *oxyR-*C strains was compared. Larvae were maintained on woodchips in the dark at 15°C. Before being injected, they were recovered for 30 min at 37°C. Saline was used as a negative control. The *G. mellonella* were randomly grouped into four groups of 30 individuals per group. Strains were cultured overnight and then subcultured to an OD_600_ of 0.4. A total of 10 µL of bacterial inoculum (10^5^ CFU/larva) was injected into the left foreleg of each wax borer using a sterile insulin syringe (B. Braun, Germany) (Russo and MacDonald, 2020). After the challenge, larvae were placed in a sterile plate and incubated at 37°C for 7 days. The humidity, temperature, and noise throughout the entire process were all controlled. Record survival or death every 12 h (no stimulus response was recorded as dead). All experiments were done in triplicate.

### 2.9 Mouse infection model

A mouse infection model was constructed by intraperitoneal injection (IP) of bacterial cultures and used to establish a reproducible systemic infection model resembling sepsis as previously described, with some modification (Jia et al., 2024; Song et al., 2024; Lim et al., 2025). Male C57BL/6 mice aged 6 to 7 weeks were purchased from Charles River Laboratories. All animal experiments were approved by the Animal Ethics Committee of Soochow University, China. Before the challenge, the mice were acclimatized for 3 days. Six mice per group were restrained for intraperitoneal injection, and the injection sites were disinfected with 75% ethanol. WT-pSTV28, *ΔoxyR*-pSTV28, and *oxyR-*C cultures were diluted with PBS to 10^4^ CFU in 100 μL and injected using a 27G needle. For the survival curve, the mortality was monitored per 24 h up to 7 days. The weights of mice were also monitored and recorded every day, and were euthanized upon assessment of humane endpoint (weight loss of more than 20% body weight). For the quantification of bacterial loads in the mice organs, mice were sacrificed at 12 hours post-infection, and blood, liver, lung, and spleen were collected. Liver, lung, and spleen were weighed and homogenized in 300 μL PBS. Bacterial burdens at each site were calculated using quantitative plating.

### 2.10 Identification of OxyR ortholog

The OxyR ortholog in *K*. *pneumoniae* ATCC 43816 was identified by BLASTP analysis using the OxyR amino acid sequence of *E*. *coli* K-12 as a query. Homologous sequences were aligned using ClustalW. The genome sequence of ATCC 43816 was retrieved from NCBI (accession GCF_016071735.1). No additional sequencing was performed in this study.

### 2.11 Statistical analyses

Experimental data were analyzed for significance via GraphPad Prism 9.5 (GraphPad Software, San Diego, California, USA). For comparisons among three groups, the Kruskal-Wallis test followed by Dunn’s multiple comparisons test was used, as the data did not fully meet the assumptions of parametric analysis. A log-rank (Mantel-Cox) test was used for the analysis of the Kaplan-Meier survival curves. *p* < 0.05 was considered indicative of statistical significance (* *p* < 0.05; ** *p* < 0.01; *** *p* < 0.001; **** *p* < 0.0001).

## 3 Results

### 3.1 VK055_RS16305 is an OxyR ortholog in *K. pneumoniae* and does not influence bacterial growth or morphological characteristics

**Figure 1:**
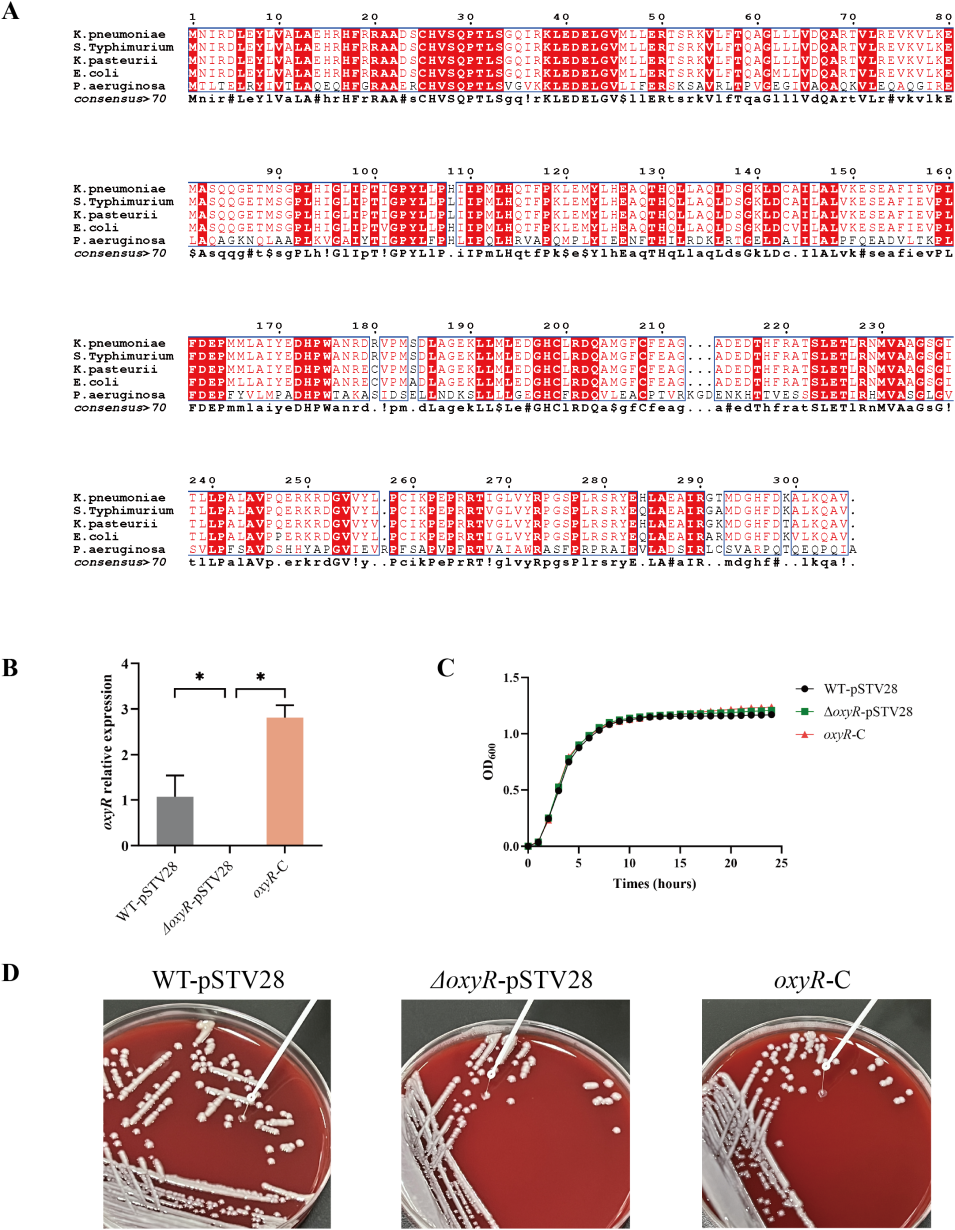
Sequence analysis of OxyR from *K*. *pneumoniae* ATCC 43816. **(A)** Alignment of VK055_RS16305 with OxyRs from *S.* Typhimurium*, K. pasteyrii, E. coli*, and *P. aeruginosa*. The conserved sequences are colored red. **(B)** Verification of the *oxyR* mutant and complement strain through qRT-PCR. Data are presented as mean ± SD. The Kruskal-Wallis test followed by Dunn’s multiple comparisons test was used to analyze qPCR data, *p < 0.05. **(C)** The deletion and complementation of *oxyR* didn’t influence the bacterial growth in LB broth. The Growth curve was determined by measuring the optical density (OD_600_) every hour throughout 24 h. The analysis of OD_600_ values at endpoint and AUC of the growth curve was presented in **Supplementary Material Figure S1**. **(D)** Colony morphology of WT-pSTV28, *ΔoxyR*-pSTV28, and *oxyR*-C.

OxyR is a widespread oxidative stress-related regulator in gram-negative bacteria. Still, little is known about its role in *K. pneumoniae*, even though it is one of the most common clinical pathogens. Here, we identified an OxyR ortholog, VK055_RS16305, in *K. pneumoniae* ATCC 43816. Genomic analysis revealed that the amino acid sequence of VK055_RS16305 was more than 95% similar to that of the OxyR homologue in *S*. Typhimurium, *K*. *pasteyrii*, and *E*. coli, whereas identity to *P. aeruginosa* OxyR was only 37% (**Figure 1A**). OxyR has been reported to protect several bacteria from oxidative damage. To assess the biological role of OxyR in *K. pneumoniae*, we constructed an *oxyR* mutant using the CRISPR-Cas9 system and a corresponding complement strain via the pSTV28 vector. qRT–PCR was used to validate the constructs and revealed that the transcription level of *oxyR* sharply decreased in *ΔoxyR*-pSTV28 compared with WT-pSTV28 and was significantly restored in *oxyR-*C, indicating successful generation of the mutant and complement strain (**Figure 1B**). The colony morphologies of WT-pSTV28, *ΔoxyR*-pSTV28, and *oxyR-*C were similar on blood agar plates (**Figure 1D**). Furthermore, there was no significant difference in the growth curve in LB broth between WT-pSTV28 and *ΔoxyR*-pSTV28 (**Figure 1C**, **Supplementary Figure S1**). These data showed that the deletion or complementation of OxyR did not influence growth or colony morphology in normal culture media.

### 3.2 OxyR plays an essential role in the antioxidant stress response of *K. pneumoniae* ATCC 43816

**Figure 2:**
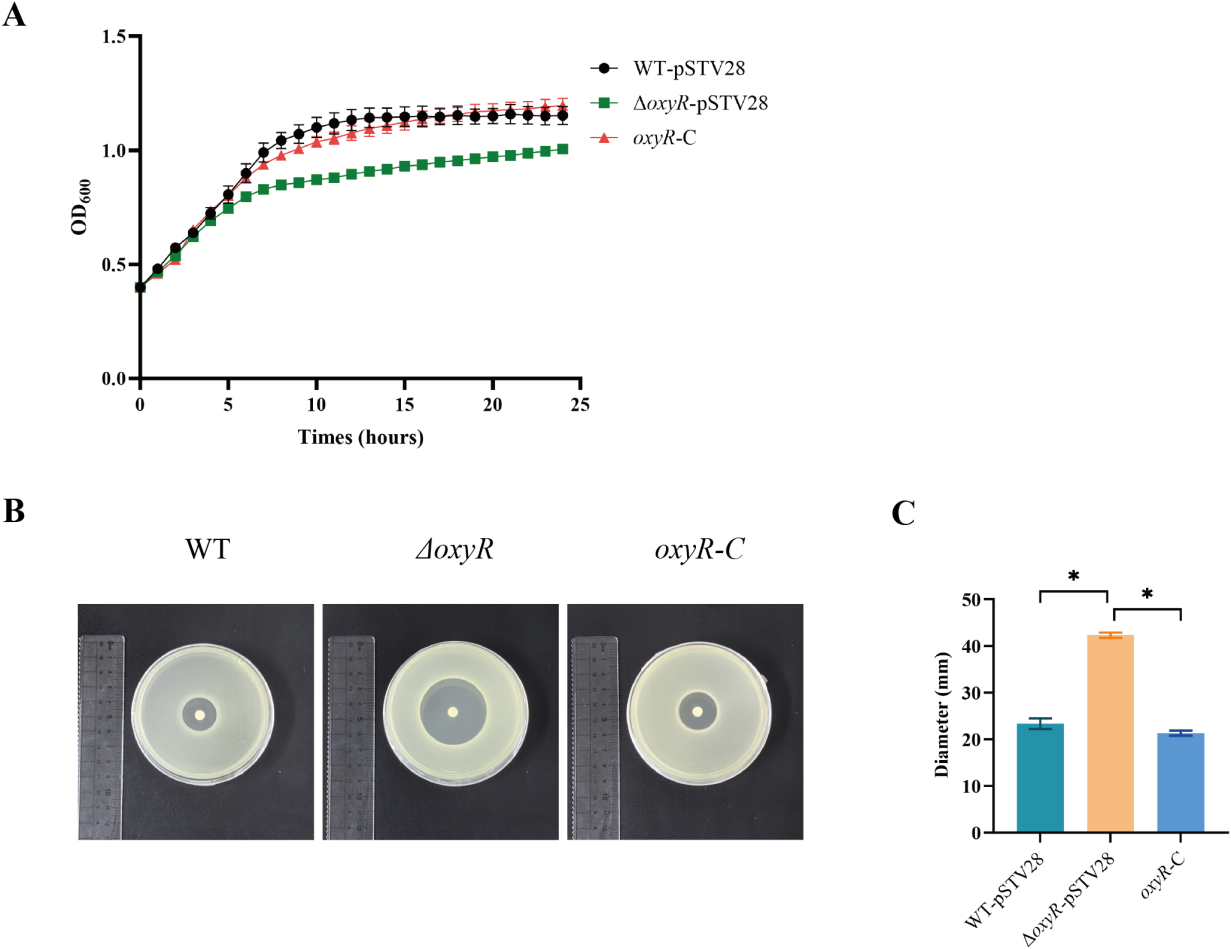
The effect of OxyR on *K*. *pneumoniae* ATCC 43816 against oxidative stress. **(A)** Growth curves of *K*. *pneumoniae* ATCC 43816 derivatives under oxidative stress (2 mM H_2_O_2_). The analysis of OD_600_ value at endpoint and AUC of the growth curve was presented in **Supplementary Material Figure S1**. **(B)** H_2_O_2_ sensitivities of *K*. *pneumoniae* ATCC 43816 derivatives were tested using a disk diffusion assay. The tolerance of the strains to H_2_O_2_ was estimated by measuring the diameters of the inhibition zones after 24 h of incubation. **(C)** Analysis of the H_2_O_2_ sensitivity test results. Data are shown as mean ± SD. The Kruskal-Wallis test followed by Dunn’s multiple comparisons test was used for comparisons, *p < 0.05.

Sensitivity tests and growth curves were used to evaluate the role of OxyR in the antioxidant stress response of *K*. *pneumoniae* ATCC 43816. From the growth curves, the endpoint OD_600_ and area under curve (AUC) of *ΔoxyR*-pSTV28 were decreased as compared to WT-pSTV28, and recovered in *oxyR*-C group. These results revealed that, compared with WT-pSTV28, *ΔoxyR*-pSTV28 was more susceptible to H_2_O_2_-mediated damage and thus grew more slowly. The OxyR complement strain *oxyR-C* exhibited restored capacity to combat oxidative stress (**Figure 2A**, **Supplementary Figure S1**). The H_2_O_2_ sensitivity tests showed the diameters of the zones of inhibition of WT-pSTV28 and *oxyR*-C were significantly smaller than that of *ΔoxyR*-pSTV28, which implies that the tolerance of *ΔoxyR*-pSTV28 to H_2_O_2_ was reduced considerably (**Figures 2B, C**).

### 3.3 Gene expression profile of *ΔoxyR* compared to WT

**Figure 3:**
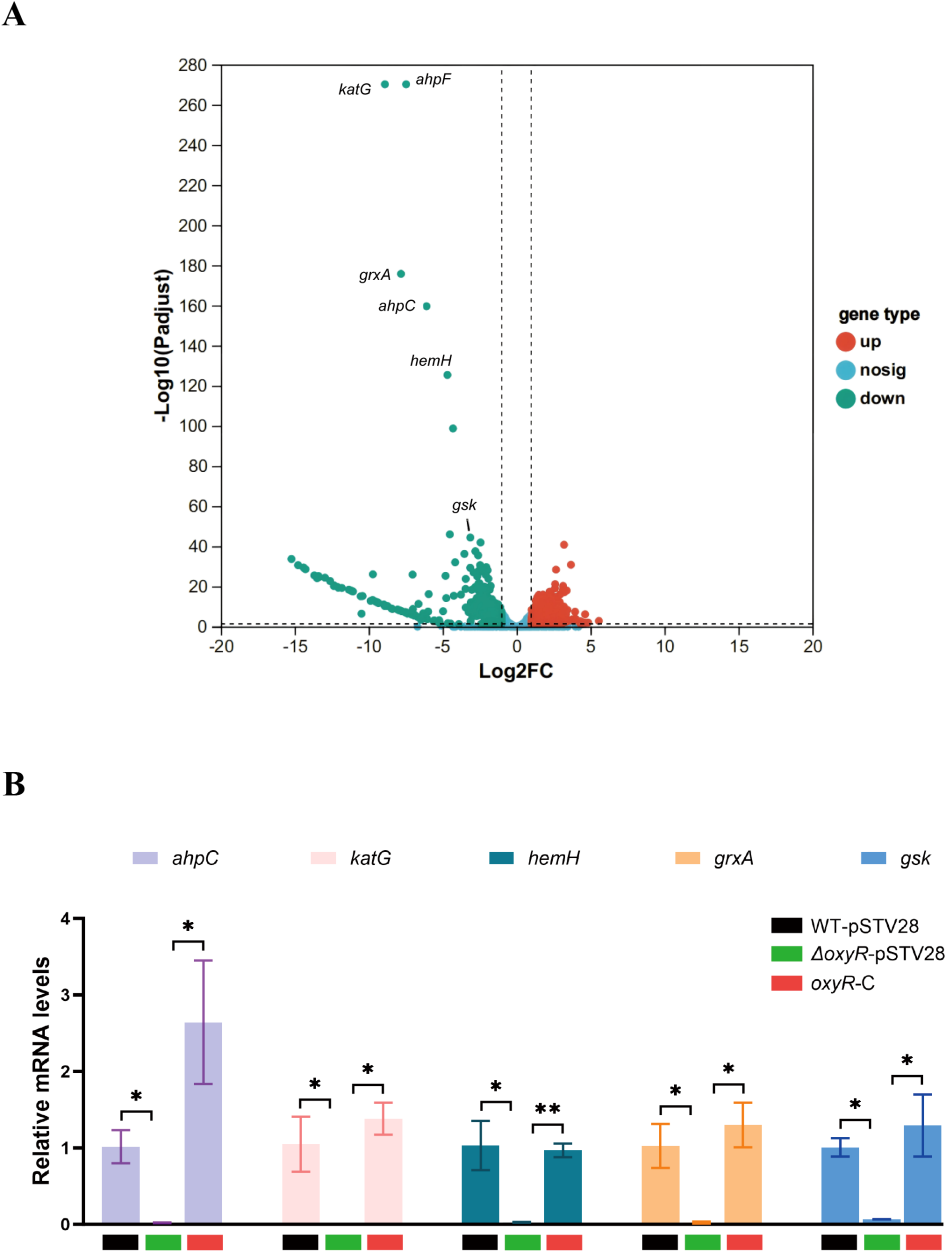
Analysis of RNA-seq data. **(A)** Volcano plot showing differentially expressed genes between WT and *ΔoxyR* induced by 1 mM H_2_O_2_ for 30 min. Orange dots represent significantly upregulated genes, and cyan dots represent significantly downregulated genes. Genes with |log_2_FC| ≥ 2 (fold change ≥ 4) and adjusted *p* < 0.05 were considered differentially expressed. **(B)** Relative mRNA levels of selected genes. Total RNA was extracted from WT-pSTV28, *ΔoxyR*-pSTV28, and *oxyR-*C after H_2_O_2_ treatment. The internal reference was 16S rRNA. Data are presented as mean ± SD. The Kruskal-Wallis test followed by Dunn’s multiple comparisons test was used to analyze qPCR data, *p < 0.05, **p < 0.01.

As a global regulator in other bacterial species, OxyR drives numerous regulons to achieve its biological function. To investigate the possible transcriptomic contributions of OxyR to oxidative defenses in *K. pneumoniae* ATCC 43816, we analyzed the gene expression profiles of the WT and *ΔoxyR* strains, both of which were stimulated with 1 mM H_2_O_2_. As shown in the volcano plot, the RNA-seq analysis revealed that 98 genes were downregulated and 40 genes were upregulated in *ΔoxyR* (**Figure 3A**, **Supplementary Table S3**). Among these genes, *katG*, *ahpC*, *hemH*, *grxA*, and *gsk* were significantly downregulated. WT-pSTV28, *ΔoxyR*-pSTV28, and *oxyR-C* were used and treated with 1 mM H_2_O_2_ to verify the differences in the expression of these selected genes. The results of qRT-PCR indicated that the mRNA levels of *katG*, *ahpC*, *hemH*, *grxA*, and *gsk* were significantly lower in *ΔoxyR*-pSTV28 than in WT-pSTV28, which was consistent with the RNA-seq results. Furthermore, the mRNA levels were restored in the complement strain *oxyR-*C (**Figure 3**).

### 3.4 Absence of *oxyR* reduces biofilm formation

**Figure 4:**
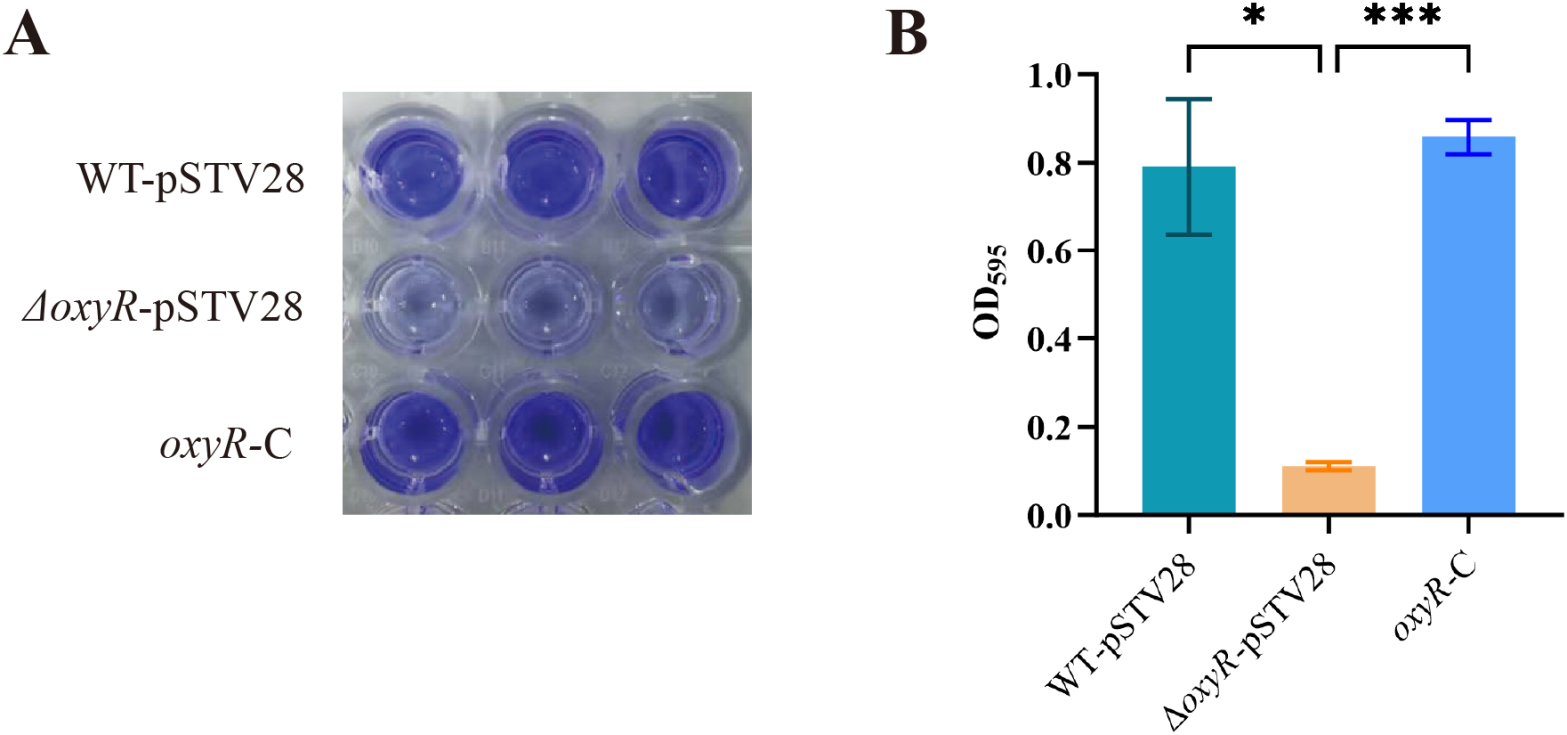
Detection of the biofilm formation abilities of WT-pSTV28, *ΔoxyR*-pSTV28, and *oxyR-*C. **(A)** Images of biofilm staining. **(B)** The extracted stain was quantified by measuring the OD_595_ to evaluate the amount of biofilm formed. Data are presented as mean ± SD. The Kruskal-Wallis test followed by Dunn’s multiple comparisons test was used to analyze data, *p < 0.05, ***p < 0.001.

Biofilm is a state of growth in which many microorganisms work together to resist external factors and survive exposure to external stimuli or a hostile environment (Guerra et al., 2022). Next, we investigated the impact of OxyR on biofilm formation. Crystal violet (CV) staining showed that the *ΔoxyR*-pSTV28 had a lighter staining than WT-pSTV28, while the *oxyR-C* recovered the staining (**Figure 4A**). Biofilm quantification showed that the biofilm formed by *ΔoxyR*-pSTV28 was almost 1/8 of that formed by WT-pSTV28. Compared with the *ΔoxyR*-pSTV28 strain, the *oxyR*-C strain exhibited increased biofilm formation (**Figure 4B**). The above results indicate that the deletion of OxyR impaired the biofilm formation of ATCC 43816.

### 3.5 Deletion of *oxyR* attenuates the *in vivo* virulence of *K. pneumoniae* ATCC 43816

**Figure 5:**
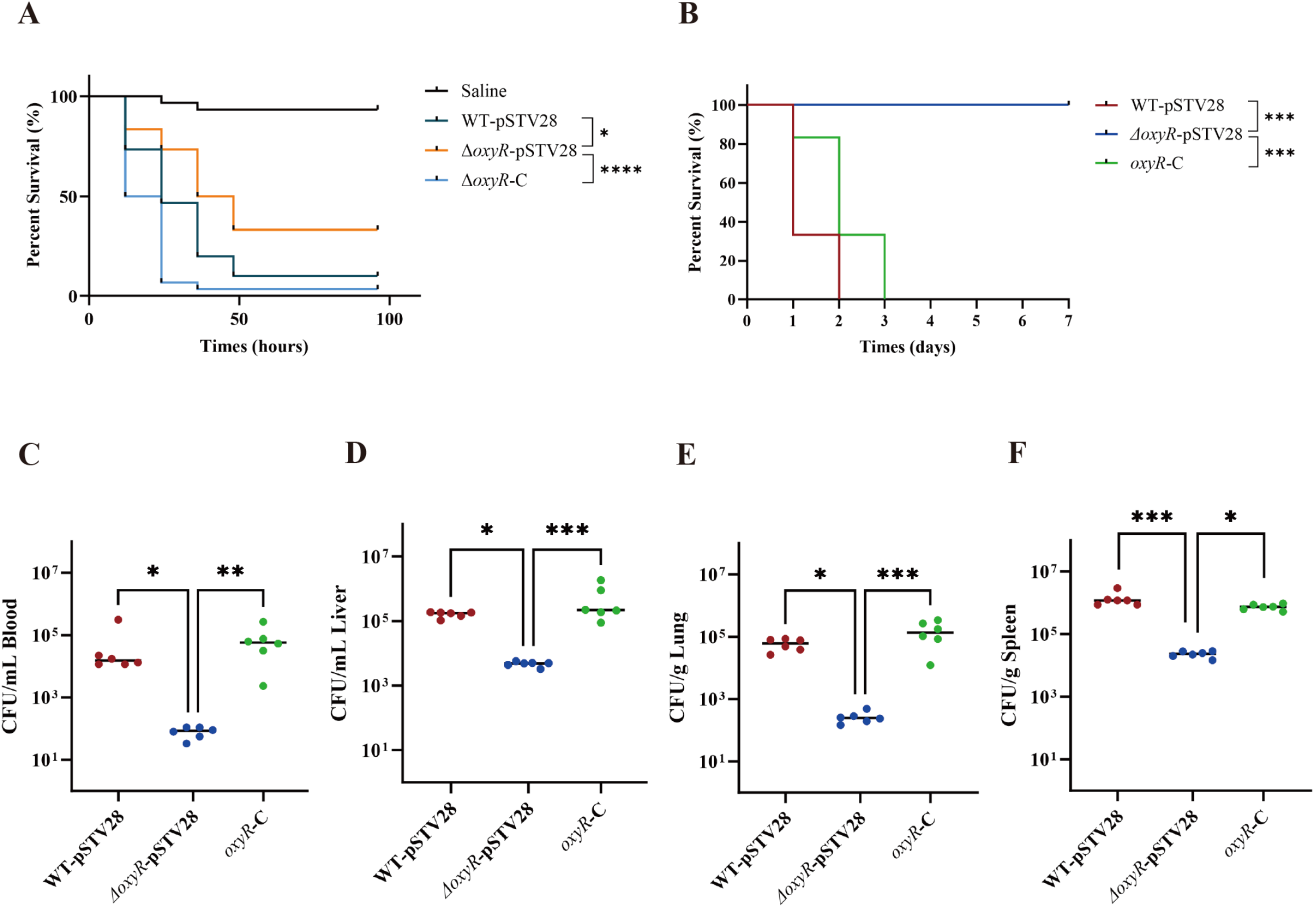
OxyR promotes the *in vivo* virulence of *K*. *pneumoniae* ATCC 43816. **(A)** Mortality of *Galleria mellonella* infection model after challenge with 10^5^ CFU of WT-pSTV28, *ΔoxyR*-pSTV28, and *oxyR*-C. n=30 for each strain group. **(B)** The virulence of WT-pSTV28, *ΔoxyR*-pSTV28, and *oxyR*-C was further evaluated in the mouse infection model by intraperitoneal injection of 10^4^ CFU bacteria. n=6 for each strain group. Survival curves were performed using the log-rank (Mantel-Cox) test. **(C-F)** Male mice were challenged with WT-pSTV28, *ΔoxyR*-pSTV28, and *oxyR*-C via intraperitoneal injection, and the CFU burden in the blood, liver, lung, and spleen was enumerated at 12 h post-infection. n=6 for each group. Each plot represents one mouse; Means are represented, and the Kruskal-Wallis test followed by Dunn’s multiple comparisons test was used to analyze data. * *p* < 0.05, ** *p* < 0.01, ***p < 0.001, ****p < 0.0001.

The *G. mellonella* killing assay and the mouse infection model were used to assess the virulence of WT-pSTV28, *ΔoxyR*-pSTV28, and *oxyR*-C strain. The survival rate of *G. mellonella* injected with WT-pSTV28 was 10% after 48 h of infection. In contrast, the survival rate of *ΔoxyR*-pSTV28 was approximately 33.3%, significantly greater than that of WT-pSTV28. After the complement of OxyR, the survival rate of *oxyR-*C significantly decreased (**Figure 5A**). In the mouse infection model, after challenge with 10^4^ CFU bacteria via intraperitoneal injection, the *ΔoxyR*-pSTV28 group showed lower mortality than the WT-pSTV28 group. In contrast, the mortality of *oxyR*-C group significantly decreased compared to the *ΔoxyR*-pSTV28 group (**Figure 5B**). Consistently, the results in female mice also revealed similar survival curves. Together, whether in male or female mice, the OxyR-deficient strains caused a decrease in virulence, and upon reintroduction of OxyR, the virulence of the strains was restored.

To evaluate the distribution of tested strains in each site, the bacterial loads of blood, liver, lung, and spleen were enumerated at 12 hours post-infection. From the results of colony-forming units (CFU) counting, we found a significant decline in bacterial loads in the blood, liver, lung, and spleen of *ΔoxyR*-pSTV28 infected mice. Complementation of OxyR in the mutant rescues the drop in bacterial loads (**Figures 5C–F**). Taken together, these data support that *oxyR* is a critical factor in mediating disseminated infection by ATCC 43816.

## 4 Discussion

In this study, we investigated the effects of *oxyR* on the oxidative tolerance and virulence of *K. pneumoniae* ATCC 43816. The results demonstrated that the growth of the *oxyR* mutant strain and its ability to tolerate H_2_O_2_ were impaired under oxidative stress. Under H_2_O_2_ stimulation, *ΔoxyR* growth was significantly inhibited, and the circular inhibition zone was larger than that of the WT. After OxyR complementation, the oxidative stress tolerance was restored in *oxyR*-C. These results indicate that OxyR is essential to defense against oxidative stress in *K. pneumoniae* ATCC 43816. This observation aligns with the previously established antioxidant role of OxyR across bacterial species (Anand et al., 2020; Méndez et al., 2022; Liu et al., 2024). In addition, a previous study reported that the deletion of *oxyR* impaired CPS production and influenced the phenotype of *K. pneumoniae* NTUH K-2044 (Srinivasan et al., 2013). However, our data indicated that neither deletion nor complementation of OxyR caused significant changes in the colony phenotype. We performed a *G. mellonella* killing assay and a mouse infection model to explore the effect of *oxyR* on the virulence of *K. pneumoniae* ATCC 43816.

Our findings are also in line with those reported by Hennequin et al (Hennequin and Forestier, 2009), who highlighted the importance of oxidative stress-related mechanisms in the pathogenicity of *K. pneumoniae*. Similar to their observations, our data support the notion that impairment of oxidative stress defenses leads to attenuated virulence. In addition, our study extends these findings by identifying OxyR as a central regulator in the hypervirulent ATCC 43816 strain, linking OxyR not only to oxidative stress tolerance but also to biofilm formation and disseminated infection, and by revealing specific downstream genes that are regulated by OxyR under oxidative stress.

Survival curves demonstrated that the deletion of *oxyR* attenuated the virulence of *K. pneumoniae* ATCC 43816 in the *G. mellonella* infection model. It should be noted that a small number of deaths occurred in the saline control group, a phenomenon also observed in previous studies using the *G. mellonella* infection model (Aperis et al., 2007). Such background mortality is generally attributed to injection-related trauma or intrinsic larval variability and does not influence the overall virulence assessment. Although this invertebrate was widely used to evaluate bacterial virulence, it has inherent limitations, such as the absence of adaptive immunity and the use of a non-natural route of infection. Thomas et al. argued that the *G. mellonella* model alone may not accurately reflect the hypervirulent phenotype of *K. pneumoniae* (Russo and Marr, 2019). Therefore, we further employed a mouse infection model to verify these findings.

In the mouse infection model, established via intraperitoneal injection, the deletion of OxyR significantly decreased the mouse mortality and bacterial loads in blood, liver, lung, and spleen. These *in vivo* results support the notion that OxyR functions as a virulence-associated factor in *K. pneumoniae*. Interestingly, the bacterial burdens in mice organs revealed that *ΔoxyR*-pSTV28 was present at a lower level in the blood (~10^1.9^ CFU/mL) and lung (~10^2.4^ CFU/g), but there were considerable amounts in the liver (~10^3.7^ CFU/g) and spleen (~10^4.4^ CFU/g). As a “firewall” or “filter”, the liver and spleen cooperate and are responsible for the clearance of invading bacteria from the blood circulation. The data on bacterial loads indicated that the *ΔoxyR*-pSTV28 strain may be captured and sequestered by the liver and spleen, thereby limiting its dissemination in the bloodstream and reducing colonization of other organs such as the lung. This finding highlights the need for further investigation into the mechanisms by which OxyR enables *K*. *pneumoniae* to evade immune clearance by the liver and spleen.

ATCC 43816 (ST493/KL2) was selected in this study because it is a well-characterized hypervirulent *K. pneumoniae* reference strain that has been widely used in pathogenesis and virulence studies. This strain displays robust virulence in both invertebrate and mammalian infection models, making it suitable for dissecting the contribution of specific regulators such as OxyR to oxidative stress tolerance and *in vivo* pathogenicity. Although our data clearly demonstrate a key role for OxyR in ATCC 43816, further work will be required to determine whether similar mechanisms operate in other hypervirulent lineages, such as ST23. Comparative studies across different hvKP clones will help to assess the generalizability of our findings.

Biofilms are among the major contributors to the virulence of *K. pneumoniae* ATCC 43816. Moreover, bacteria with more potent biofilm formation abilities more easily colonize and establish niches in the host (Vuotto et al., 2017; Guerra et al., 2022; Karami-Zarandi et al., 2023; Li and Ni, 2023). Beyond its antioxidant function, OxyR has been implicated in biofilm formation in diverse pathogens (Shanks et al., 2007; Srinivasan et al., 2013). In the present work, the data from the biofilm formation assay indicated that *oxyR* positively influenced biofilm formation in *K. pneumoniae* ATCC 43816.

As a global transcriptional regulator, OxyR controls numerous genes involved in diverse biological processes. RNA-seq analysis revealed that *hemH*, *grxA*, *gsk*, *katG*, and *ahpC* were significantly downregulated in the *ΔoxyR* strain compared to WT. In a previous study, the gene *hemH* was reported as a member of the *E. coli* OxyR regulon (Zheng et al., 2001). Its product, HemH, can catalyze the final step of heme biosynthesis and promote the insertion of ferrous iron into protoporphyrin IX (Mancini and Imlay, 2015). KatG is a heme catalase that scavenges endogenous and exogenous H_2_O_2_ and catalyzes the decomposition of hydrogen peroxide to water and molecular oxygen (Hébrard et al., 2009). The absence of *katG* in *Salmonella* reduces bacterial adhesion and invasion of macrophages and leads to the accumulation of intracellular ROS (Kirthika et al., 2022). The catalytic efficiency of *ahpC* was reported to be better than that of the catalases above; thus, it is crucial in combating the oxidative stress induced by lower levels of H_2_O_2_ (Ma and Payne, 2012; Kamariah et al., 2018; Nelson et al., 2018). In *E. coli*, *ahpC* detoxifies peroxides, and deletion of *ahpC* leads to increased sensitivity to iron chelation (Parsonage et al., 2008; Ma and Payne, 2012). In addition, Grx acts as an electron donor and regulator of cellular function in response to oxidative stress. *grxA* is known to activate the expression of several antioxidant defensive genes in response to elevated levels of H_2_O_2_ (Fernandes and Holmgren, 2004). Gsk is the salvage enzyme that feeds inosine and guanosine into the purine synthesis pathway. This means that *gsk* can help bacteria such as *E. coli*, capable of growing in many environments, adapt to stress/starvation and then recover from stress/starvation (Petchiappan and Gottesman, 2020; Wang et al., 2020). In the present study, the results of RNA-seq analysis and qRT-PCR demonstrated that these oxidative stress-related genes are downregulated with *oxyR* deletion, suggesting that OxyR may promote the capacity to prevent oxidative stress through the activation of *hemH*, *grxA*, *gsk*, *katG*, and *ahpC* in *K. pneumoniae* ATCC 43816.

This study has certain limitations. Further research is needed to elucidate the detailed molecular mechanism by which OxyR regulates these downstream genes and contributes to virulence. Moreover, additional studies focusing on the interactions between OxyR and host immune responses may provide deeper insights into the role of this regulator in the pathogenesis of *K. pneumoniae* ATCC 43816.

Overall, our findings reveal that OxyR is crucial for oxidative stress defense, biofilm formation, and virulence of *K. pneumoniae*, providing a basis for future studies targeting OxyR-mediated pathways.

## Data Availability

The datasets presented in this study can be found in online repositories. The names of the repository/repositories and accession number(s) can be found in the article/**Supplementary Material**.
