# Supplementary material for: OxyR contributes to the oxidative stress capacity and virulence of hypervirulent Klebsiella pneumoniae ATCC 43816
Source: Front Cell Infect Microbiol. 2026 Jan 7;15:1661384. doi: 10.3389/fcimb.2025.1661384 (PMC12819678; doi:10.3389/fcimb.2025.1661384)
Supplement: Supplementary file 1 [file DataSheet1.pdf]

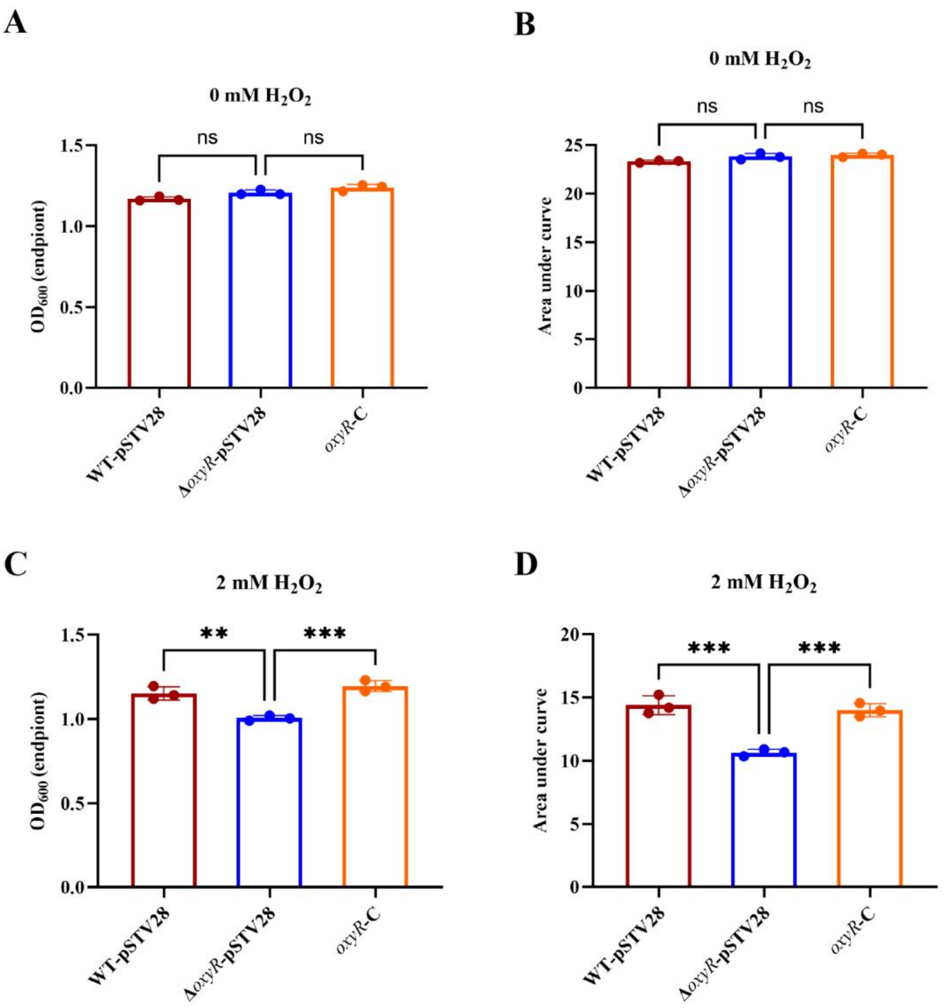

Figure S1. The OD_{600} values at endpoint and AUC of growth curves were analyzed to compare the growth curves of WT-pSTV28, *ΔoxyR*-pSTV28, and *oxyR*-C. (A-B) There were no significant difference of OD_{600} values and AUC of growth curves between three stains under LB without H_2O_2. *oxyR* mutant showed decreased OD_{600} value at endpoint **(C)** and area under curve **(D)**. Data are shown as mean ± SD, the Kruskal-Wallis test followed by Dunn's multiple comparisons test was used to analyze data, ** $p < 0.01$; *** $p < 0.001$; ns, no significant.

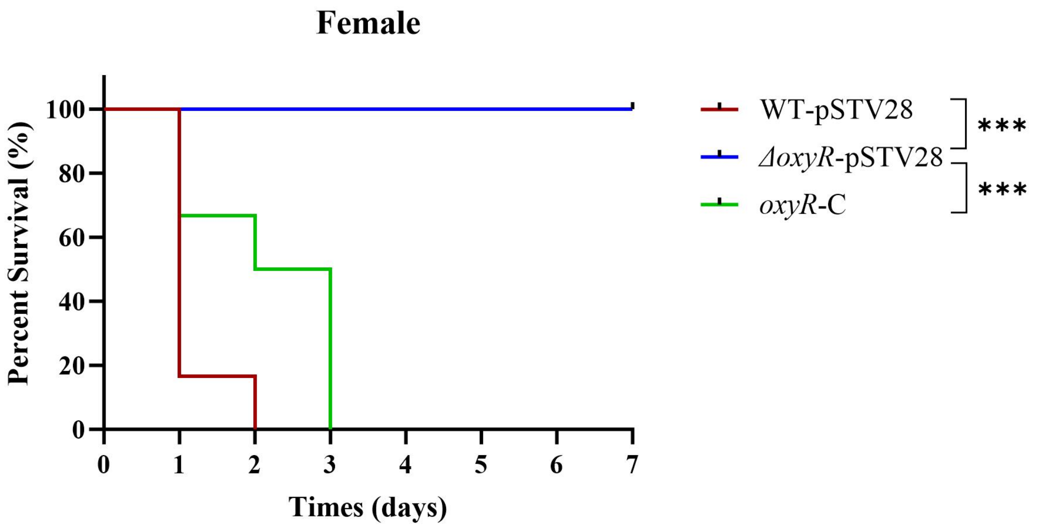

Figure S2. Survival curve of female mice after intraperitoneal injection with 10^4 CFU WT-pSTV28, *ΔoxyR*-pSTV28, and *oxyR*-C. n=6 for each strain group. Log-rank (Mantel-Cox) test was performed to compare the survival curves. *** $p < 0.001$.

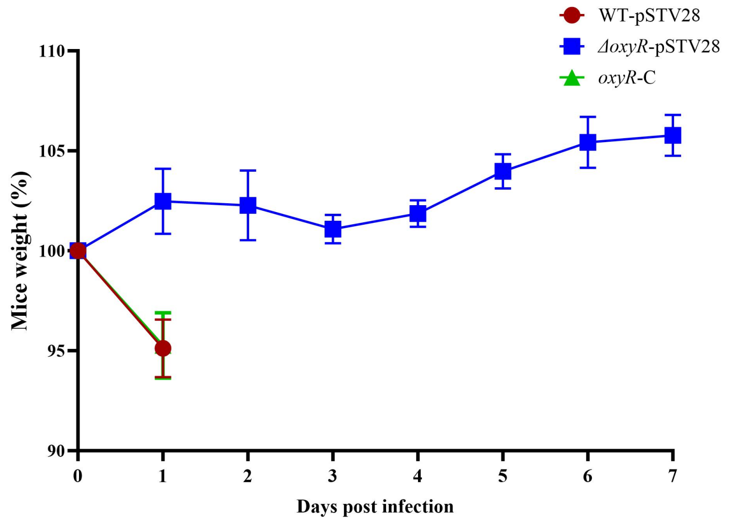

Figure S3. Evalution of of body weight change during infection. Male mice were challenge with 10^4 CFU WT-pSTV28, *ΔoxyR*-pSTV28, and *oxyR*-C. Data are shown as mean ± SD. n=6 for each group.
