# Supplementary material for: OxyR contributes to the oxidative stress capacity and virulence of hypervirulent Klebsiella pneumoniae ATCC 43816
Source: Front Cell Infect Microbiol. 2026 Jan 7;15:1661384. doi: 10.3389/fcimb.2025.1661384 (PMC12819678; doi:10.3389/fcimb.2025.1661384)
Supplement: Supplementary file 2 [file Table1.docx]

| **Table S1 Oligonucleotide** **primers used in this study** | | |
| --- | --- | --- |
| **Target** | **Primers (Forward/Reverse,5’→ 3’)** | **Source/reference/function** |
| **Construction of mutant** | | |
| N20-F | tagtCCGCAGGAGAGAAAACGCGA | Construct pSGKP with targeted sgRNA (pSGKP-*oxyR*-N20) |
| N20-R | aaactcgcgttttctctcctgcgg |  |
| M13-Rev | CAGGAAACAGCTATGACC |  |
| *oxyR*-1 | ggagacgctgagtacgacag | Construct a homologous recombination arm as the *oxyR*-deletion repair template |
| *oxyR*-2 | ttacgcagcgagttctgtcagctctttatt |  |
| *oxyR*-3 | tgacagaactcgctgcgtaaaaaactgaaa |  |
| *oxyR*-4 | gctgtgagttttccagtgat |  |
| **Construction of complemented mutant** | | |
| *oxyR*-CF | CGGAATTCatgaatattcgcgatcttga | Construct the recombinant plasmid pSTV28-*oxyR* |
| *oxyR*-CR | cggAAGCTTttaaaccgcctgttttaacg |  |
| **qPCR** | | |
| qRT-*oxyR*-For | GAAGCTGGAAATGTATCT | Primers used for quantitative reverse-transcription PCR (qRT-PCR) |
| qRT-*oxyR*-Rev | TTCATAGATAGCCAACAT |  |
| qRT-*hemH*-For | CGAAACGCTGGAAGAAAT |  |
| qRT-*hemH*-Rev | TTGACCATCATCTCAATATGC |  |
| qRT-*grxA*-For | GGCTGCCCTTACTGCGTT |  |
| qRT-*grxA*-Rev | GCCCAAGCTTCAAAATCA |  |
| qRT-*gsk*-For | ATGAAGGCTATTGAGTATGC |  |
| qRT-*gsk*-Rev | CATTGCCAGAATAGAGACAT |  |
| qRT-*katG*-For | GTTAAACCAGCACTCCAATC |  |
| qRT-*katG*-Rev | AACAAGCCAATATAGCTACC |  |
| qRT-*aphC*-For | tgaccattacgaagaact |  |
| qRT-*aphC*-Rev | tcatcgcatatttgattttg |  |
| 16S-For | CTACAAGACTCTAGCCTGCCAGTTTC |  |
| 16S-Rev | GCGGTCTGTCAAGTCGGATGTG |  |
