# Supplementary material for: OxyR contributes to the oxidative stress capacity and virulence of hypervirulent Klebsiella pneumoniae ATCC 43816
Source: Front Cell Infect Microbiol. 2026 Jan 7;15:1661384. doi: 10.3389/fcimb.2025.1661384 (PMC12819678; doi:10.3389/fcimb.2025.1661384)
Supplement: Supplementary file 3 [file Table2.docx]

| **Table S2 Strains and plasmids used in this study** | | |
| --- | --- | --- |
| **Target** | **Description** | **Source/reference/function** |
| **Strains** |  |  |
| *E. coli* DH5α |  | Laboratory stock |
| 1. *pneumoniae* ATCC43816 | Wild type | American Type Culture Collection |
| *ΔoxyR* | ATCC43816 *oxyR*-deletion mutant | This study |
| WT-pSTV28 | ATCC43816 with the empty vector pSTV28, Cm^R^ | This study |
| *ΔoxyR*-pSTV28 | *ΔoxyR* with the empty vector pSTV28, Cm^R^ | This study |
| *ΔoxyR-*C | *ΔoxyR* with the complement plasmid pSTV28-*oxyR*, Cm^R^ | This study |
| **Plasmids** | | |
| pCasKP | Temperature sensitive, Apr^r^ | Laboratory stock |
| pSGKP | Rif^r^ | Laboratory stock |
| pSGKP-*oxyR*-N20 | pSGKP with the *oxyR* gene | This study |
| pSTV28 | Cm^R^ | Takara |
| pSTV28-*oxyR* | pSTV28 with the *oxyR* gene | This study |
