# Supplementary material for: OxyR contributes to the oxidative stress capacity and virulence of hypervirulent Klebsiella pneumoniae ATCC 43816
Source: Front Cell Infect Microbiol. 2026 Jan 7;15:1661384. doi: 10.3389/fcimb.2025.1661384 (PMC12819678; doi:10.3389/fcimb.2025.1661384)
Supplement: Supplementary file 4 [file Table3.docx]

| **Table S3 Differentially expressed genes in *ΔoxyR* relative to the wild-type strain ATCC43816 after stimulation of H_2_O_2_** | | | |
| --- | --- | --- | --- |
| Gene_id | Gene name | Gene description | Log_2_FC |
| VK055_RS03375 | katG | catalase/peroxidase HPI | -8.895906521 |
| VK055_RS08040 | VK055_RS08040 | GrxA family glutaredoxin | -7.808260163 |
| VK055_RS09425 | ahpF | alkyl hydroperoxide reductase subunit F | -7.46179503 |
| VK055_RS09430 | ahpC | alkyl hydroperoxide reductase subunit C | -6.082757191 |
| VK055_RS11020 | VK055_RS11020 | PTS sugar transporter subunit IIA | -5.974324219 |
| VK055_RS06540 | VK055_RS06540 | EAL domain-containing protein | -4.751175623 |
| VK055_RS10480 | hemH | ferrochelatase | -4.671404165 |
| VK055_RS09555 | mtnK | S-methyl-5-thioribose kinase | -4.504789266 |
| VK055_RS16650 | glnA | glutamate--ammonia ligase | -4.291832992 |
| VK055_RS08415 | mntS | manganase accumulation protein MntS | -4.237195711 |
| VK055_RS14245 | VK055_RS14245 | PRD domain-containing protein | -4.147607176 |
| VK055_RS08045 | ybjM | inner membrane protein YbjM | -3.756902428 |
| VK055_RS12895 | yaaA | peroxide stress protein YaaA | -3.524882398 |
| VK055_RS05925 | VK055_RS05925 | YmiA family putative membrane protein | -3.434954028 |
| VK055_RS14445 | rpsF | 30S ribosomal protein S6 | -3.44592331 |
| VK055_RS21465 | mrkA | type 3 fimbria major subunit MrkA | -3.43733247 |
| VK055_RS14240 | VK055_RS14240 | SFCGS family glycine-rich protein | -3.277250718 |
| VK055_RS21130 | VK055_RS21130 | LuxR C-terminal-related transcriptional regulator | -3.235359438 |
| VK055_RS14235 | VK055_RS14235 | DUF4312 family protein | -3.236636202 |
| VK055_RS28285 | ypfM | protein YpfM | -3.129909975 |
| VK055_RS10475 | VK055_RS10475 | inosine/guanosine kinase | -3.125219435 |
| VK055_RS04820 | VK055_RS04820 | anion permease | -3.107231611 |
| VK055_RS15690 | VK055_RS15690 | PTS sugar transporter subunit IIB | -3.078098347 |
| VK055_RS05420 | VK055_RS05420 | MBL fold metallo-hydrolase | -3.017982349 |
| VK055_RS25035 | bglG | transcriptional antiterminator BglG | -2.931233841 |
| VK055_RS18000 | VK055_RS18000 | hypothetical protein | -2.905853322 |
| VK055_RS13230 | VK055_RS13230 | GntR family transcriptional regulator | -2.847931716 |
| VK055_RS00045 | VK055_RS00045 | hypothetical protein | -2.845008566 |
| VK055_RS06415 | VK055_RS06415 | translesion error-prone DNA polymerase V autoproteolytic subunit | -2.841233337 |
| VK055_RS15915 | metR | HTH-type transcriptional regulator MetR | -2.791347543 |
| VK055_RS24975 | folE | GTP cyclohydrolase I FolE | -2.78371943 |
| VK055_RS22135 | VK055_RS22135 | HAAAP family serine/threonine permease | -2.756371855 |
| VK055_RS03775 | VK055_RS03775 | PTS sugar transporter subunit IIA | -2.688335064 |
| VK055_RS22235 | queD | 6-carboxytetrahydropterin synthase QueD | -2.684394608 |
| VK055_RS09460 | mtnC | acireductone synthase | -2.653782437 |
| VK055_RS11010 | VK055_RS11010 | peroxiredoxin | -2.628122604 |
| VK055_RS20310 | VK055_RS20310 | TIGR04211 family SH3 domain-containing protein | -2.627804915 |
| VK055_RS00390 | zwf | glucose-6-phosphate dehydrogenase | -2.594919582 |
| VK055_RS09455 | VK055_RS09455 | pyridoxal phosphate-dependent aminotransferase | -2.573685947 |
| VK055_RS14440 | priB | primosomal replication protein N | -2.546498054 |
| VK055_RS10315 | VK055_RS10315 | hypothetical protein | -2.551303628 |
| VK055_RS18640 | glpE | thiosulfate sulfurtransferase GlpE | -2.536945203 |
| VK055_RS14435 | rpsR | 30S ribosomal protein S18 | -2.518862626 |
| VK055_RS14230 | VK055_RS14230 | DUF4311 domain-containing protein | -2.526977215 |
| VK055_RS08445 | glnH | glutamine ABC transporter substrate-binding protein GlnH | -2.510201743 |
| VK055_RS06220 | xthA | exodeoxyribonuclease III | -2.487041664 |
| VK055_RS18655 | malT | HTH-type transcriptional regulator MalT | -2.482137425 |
| VK055_RS23255 | VK055_RS23255 | tRNA/rRNA methyltransferase | -2.459719433 |
| VK055_RS18250 | gorA | glutathione-disulfide reductase | -2.431221878 |
| VK055_RS00665 | VK055_RS00665 | YcgL domain-containing protein | -2.400467279 |
| VK055_RS17505 | VK055_RS17505 | MIP/aquaporin family protein | -2.403442598 |
| VK055_RS13465 | VK055_RS13465 | DUF1127 domain-containing protein | -2.39830005 |
| VK055_RS24155 | VK055_RS24155 | Nramp family divalent metal transporter | -2.389882829 |
| VK055_RS18065 | VK055_RS18065 | ABC transporter substrate-binding protein | -2.380884394 |
| VK055_RS04085 | VK055_RS04085 | ParD-like family protein | -2.363444552 |
| VK055_RS12710 | carA | glutamine-hydrolyzing carbamoyl-phosphate synthase small subunit | -2.348477135 |
| VK055_RS22440 | VK055_RS22440 | metal ABC transporter substrate-binding protein | -2.351870228 |
| VK055_RS11550 | VK055_RS11550 | YjhX family toxin | -2.329836243 |
| VK055_RS18265 | prlC | oligopeptidase A | -2.304623944 |
| VK055_RS15475 | rluF | 23S rRNA pseudouridine(2604) synthase RluF | -2.291079227 |
| VK055_RS03575 | VK055_RS03575 | ASCH domain-containing protein | -2.260174904 |
| VK055_RS07175 | efeO | iron uptake system protein EfeO | -2.249767628 |
| VK055_RS06410 | VK055_RS06410 | Y-family DNA polymerase | -2.245994985 |
| VK055_RS09155 | chiP | chitoporin | -2.237502302 |
| VK055_RS25145 | VK055_RS25145 | RcnB family protein | -2.234419869 |
| VK055_RS06825 | VK055_RS06825 | TonB-dependent siderophore receptor | -2.223580005 |
| VK055_RS14505 | bsmA | biofilm peroxide resistance protein BsmA | -2.220127086 |
| VK055_RS16770 | rbsD | D-ribose pyranase | -2.181953002 |
| VK055_RS23975 | VK055_RS23975 | RpoE-regulated lipoprotein | -2.180085017 |
| VK055_RS23175 | raiA | ribosome-associated translation inhibitor RaiA | -2.168329799 |
| VK055_RS19455 | zapG | Z-ring associated protein ZapG | -2.173168891 |
| VK055_RS10270 | cadB | cadaverine/lysine antiporter | -2.169548407 |
| VK055_RS20005 | VK055_RS20005 | Gfo/Idh/MocA family oxidoreductase | -2.151158775 |
| VK055_RS06980 | rimJ | ribosomal protein S5-alanine N-acetyltransferase | -2.146907172 |
| VK055_RS12915 | thrA | bifunctional aspartate kinase/homoserine dehydrogenase I | -2.136138854 |
| VK055_RS29425 | VK055_RS29425 | hypothetical protein | -2.124323015 |
| VK055_RS00050 | VK055_RS00050 | hypothetical protein | -2.115709319 |
| VK055_RS05330 | ttcA | tRNA 2-thiocytidine(32) synthetase TtcA | -2.093379056 |
| VK055_RS15810 | hemG | menaquinone-dependent protoporphyrinogen IX dehydrogenase | -2.091969822 |
| VK055_RS22820 | nrdF | class 1b ribonucleoside-diphosphate reductase subunit beta | -2.090179822 |
| VK055_RS08455 | glnQ | glutamine ABC transporter ATP-binding protein GlnQ | -2.083872196 |
| VK055_RS16330 | ppc | phosphoenolpyruvate carboxylase | -2.077574314 |
| VK055_RS14120 | pyrI | aspartate carbamoyltransferase regulatory subunit | -2.078275742 |
| VK055_RS19280 | VK055_RS19280 | YhdT family protein | -2.069401887 |
| VK055_RS07020 | VK055_RS07020 | rhodanese-related sulfurtransferase | -2.065866817 |
| VK055_RS04445 | VK055_RS04445 | rhodanese-like domain-containing protein | -2.051622595 |
| VK055_RS18635 | glpD | glycerol-3-phosphate dehydrogenase | -2.043955937 |
| VK055_RS16155 | VK055_RS16155 | LysR family transcriptional regulator | -2.046722221 |
| VK055_RS25840 | iroN | siderophore salmochelin receptor IroN | -2.042019859 |
| VK055_RS21515 | VK055_RS21515 | multidrug/biocide efflux PACE transporter | -2.039267407 |
| VK055_RS04675 | mlc | sugar metabolism global transcriptional regulator Mlc | -2.037376447 |
| VK055_RS25985 | pmrD | signal transduction protein PmrD | -2.034425198 |
| VK055_RS04720 | kpnF | multidrug efflux SMR transporter subunit KpnF | -2.027375675 |
| VK055_RS07870 | clpS | ATP-dependent Clp protease adapter ClpS | -2.030078784 |
| VK055_RS15745 | tuf | elongation factor Tu | -2.024088295 |
| VK055_RS23740 | upp | uracil phosphoribosyltransferase | -2.020620346 |
| VK055_RS11780 | gmhB | D-glycero-beta-D-manno-heptose 1%2C7-bisphosphate 7-phosphatase | -2.001306903 |
| VK055_RS01420 | VK055_RS01420 | MetQ/NlpA family lipoprotein | -2.00204603 |
| VK055_RS19935 | VK055_RS19935 | DoxX family protein | 2.009207716 |
| VK055_RS06570 | VK055_RS06570 | manganese catalase family protein | 2.009608535 |
| VK055_RS01095 | VK055_RS01095 | gamma-glutamylcyclotransferase | 2.023473736 |
| VK055_RS17385 | VK055_RS17385 | nuclear transport factor 2 family protein | 2.023763599 |
| VK055_RS08035 | VK055_RS08035 | YbjC family protein | 2.039447284 |
| VK055_RS22620 | norR | nitric oxide reductase transcriptional regulator NorR | 2.039811352 |
| VK055_RS18230 | VK055_RS18230 | GNAT family protein | 2.046305765 |
| VK055_RS04655 | VK055_RS04655 | DmsC/YnfH family molybdoenzyme membrane anchor subunit | 2.061701968 |
| VK055_RS28555 | VK055_RS28555 | hypothetical protein | 2.113277378 |
| VK055_RS04920 | VK055_RS04920 | YdiH family protein | 2.114158853 |
| VK055_RS25020 | VK055_RS25020 | CidA/LrgA family protein | 2.133470776 |
| VK055_RS06565 | VK055_RS06565 | cytochrome ubiquinol oxidase subunit I | 2.219871183 |
| VK055_RS22380 | VK055_RS22380 | iron ABC transporter permease | 2.220205228 |
| VK055_RS15135 | VK055_RS15135 | fimbrial protein | 2.236623476 |
| VK055_RS17145 | VK055_RS17145 | GNAT family N-acetyltransferase | 2.237954697 |
| VK055_RS17375 | VK055_RS17375 | LysR family transcriptional regulator | 2.273678527 |
| VK055_RS15010 | phnN | ribose 1%2C5-bisphosphokinase | 2.283127534 |
| VK055_RS18505 | livF | high-affinity branched-chain amino acid ABC transporter ATP-binding protein LivF | 2.324944508 |
| VK055_RS12540 | VK055_RS12540 | MFS transporter | 2.325232803 |
| VK055_RS21490 | mkrJ | phosphodiesterase MrkJ | 2.328867353 |
| VK055_RS00460 | pphA | protein-serine/threonine phosphatase | 2.35413421 |
| VK055_RS08855 | VK055_RS08855 | YbgS-like family protein | 2.365618233 |
| VK055_RS01355 | VK055_RS01355 | hypothetical protein | 2.389690844 |
| VK055_RS24010 | cysA | sulfate/thiosulfate ABC transporter ATP-binding protein CysA | 2.412662383 |
| VK055_RS22895 | VK055_RS22895 | NAD(P)H-dependent oxidoreductase | 2.428643181 |
| VK055_RS17195 | VK055_RS17195 | type II toxin-antitoxin system RelE/ParE family toxin | 2.429235623 |
| VK055_RS03195 | VK055_RS03195 | hypothetical protein | 2.490876823 |
| VK055_RS10690 | VK055_RS10690 | MGMT family protein | 2.590333238 |
| VK055_RS20195 | evgA | acid-sensing system DNA-binding response regulator EvgA | 2.594923433 |
| VK055_RS10740 | VK055_RS10740 | SgrR family transcriptional regulator | 2.611081652 |
| VK055_RS21290 | VK055_RS21290 | SDR family oxidoreductase | 2.634052966 |
| VK055_RS06580 | VK055_RS06580 | ferritin-like domain-containing protein | 2.641454377 |
| VK055_RS05495 | VK055_RS05495 | hypothetical protein | 2.665449825 |
| VK055_RS08745 | VK055_RS08745 | biofilm development regulator YmgB/AriR family protein | 2.906863397 |
| VK055_RS04660 | dmsD | Tat proofreading chaperone DmsD | 2.951135865 |
| VK055_RS14370 | VK055_RS14370 | DUF1107 domain-containing protein | 3.148854735 |
| VK055_RS05055 | VK055_RS05055 | DUF1398 domain-containing protein | 3.348870742 |
| VK055_RS08750 | VK055_RS08750 | helix-turn-helix transcriptional regulator | 3.389426635 |
| VK055_RS17140 | VK055_RS17140 | DMT family transporter | 3.397018666 |
| VK055_RS01315 | VK055_RS01315 | YceI family protein | 3.683520418 |
